# Antimicrobial peptides with cell-penetrating activity as prophylactic and treatment drugs

**DOI:** 10.1042/BSR20221789

**Published:** 2022-09-23

**Authors:** Gabriel del Rio, Mario A. Trejo Perez, Carlos A. Brizuela

**Affiliations:** 1 Department of Biochemistry and Structural Biology, Instituto de Fisiologia Celular, UNAM, Mexico City 04510, Mexico; 2 Department of Computer Science, CICESE Research Center, Ensenada 22860, Mexico

**Keywords:** antimicrobial peptides, autophagy, cell penetrating peptide, machine learning, microbiome

## Abstract

Health is fundamental for the development of individuals and evolution of species. In that sense, for human societies is relevant to understand how the human body has developed molecular strategies to maintain health. In the present review, we summarize diverse evidence that support the role of peptides in this endeavor. Of particular interest to the present review are antimicrobial peptides (AMP) and cell-penetrating peptides (CPP). Different experimental evidence indicates that AMP/CPP are able to regulate autophagy, which in turn regulates the immune system response. AMP also assists in the establishment of the microbiota, which in turn is critical for different behavioral and health aspects of humans. Thus, AMP and CPP are multifunctional peptides that regulate two aspects of our bodies that are fundamental to our health: autophagy and microbiota. While it is now clear the multifunctional nature of these peptides, we are still in the early stages of the development of computational strategies aimed to assist experimentalists in identifying selective multifunctional AMP/CPP to control nonhealthy conditions. For instance, both AMP and CPP are computationally characterized as amphipatic and cationic, yet none of these features are relevant to differentiate these peptides from non-AMP or non-CPP. The present review aims to highlight current knowledge that may facilitate the development of AMP’s design tools for preventing or treating illness.

## Introduction

During the history of drug development, scientists have explored the use of small molecules (e.g., penicillin, metformine), large-size molecules (e.g., antibodies, vaccines), and more recently mid-size molecules; for instance, in recent years, the FDA for the first time approved a therapy based on antisense oligonucleotides [1] and more peptides are being approved for pharmaceutical purposes [2]. The molecular size of pharmaceutical drugs is relevant for different reasons. For instance, small molecules may not have the size to cover the area required for providing specificity in some cases [3,4], peptides may be more expensive [5], yet more effective in advanced human trials [6]. In that sense, mid-size molecules such as oligonucleotides or peptides are a new frontier in pharmaceutics that provide an intermediate solution to these problems, that is, they may have the right size to cover large areas to provide specificity and are not too large and their cost of synthesis is not so expensive. In the present review, we will focus on analyzing potential therapeutic uses of peptides. In particular, we will focus on two classes of peptides: antimicrobial peptides (AMP) and cell-penetrating peptides (CPP).

Therapeutic drugs aim to treat any condition that affects health. The concept of health has been reviewed recently [7], and it has been proposed that such condition does not represent a ‘normal’ or ‘appropriate’ state of humans or living organisms. Instead, health is defined as the capacity of living organisms for adaptive variation, consequently disease or illness should be viewed as the reduction in such capacity. From that perspective, a healthy organism should maintain homeostasis and allostasis. Homeostasis is considered a process that maintains without change the cellular processes of a living form, while allostasis refers to the stability achieved through change of its components to constantly adapt to the changing environment [8]. These concepts are well aligned with two emergent cellular mechanisms that are now recognized as fundamental to health: autophagy and microbiota. Autophagy is a catabolic process that enables cells to maintain their functionality by degrading what does not work for the cell anymore or degrade anything that is affecting the cell functionality, while the microbiota comprises cellular microorganisms that live within animal tissues to help them perform their tasks. As we will review in the present work, AMP with CPP activity are able to control both autophagy and microbiome, constituting a new class of biologically active compounds that may endow animals with the capacity to maintain allostasis and/or homeostasis, hence promote healthy states either to treat an illness or to prevent it (prophylactic). The idea that prevention is better than treatment of diseases has been thoroughly analyzed [9], and while it is true that when prevention is too expensive treatment is preferred, there are cases where humans, animals, and insects present behaviors that prefer prevention over treatment. For instance, humans have learned over many years the importance of maintaining clean spaces to prevent diseases; termites that need to share their nest with many bacteria have developed social strategies to control the spread of mortal fungal infections [10]. If these organisms have adapted their behavior to prevent diseases, is it possible that during evolution some molecular mechanisms have been selected for preventing diseases? Here, we review previous published results to support the idea that if preventing diseases could be genetically inherited, this may have occurred through AMP with CPP activity. We are still in an early stage for designing these peptides to prevent or treat complex traits such us gut microbiome dysbiosis; hence, it is an appropriate time to highlight where we stand and what to look for in the near future.

## AMP structure–activity relationship

Peptide structure may be described in different ways. For instance, peptides may be described by their amino acid sequence (primary structure), the contents of structural patterns such as β-sheets or α-helices (secondary structure), by the dispositions in the three-dimensional space of the peptide atoms (tertiary structure), by the composition of chemical groups (e.g., carboxylates, primary amines), chemical properties (e.g., polarity, isoelectric point), among other chemical descriptors (e.g., electrotopological states, entropy). There are different suites of computer programs aimed to calculate such structure descriptors of proteins and peptides [11–14]. On the other hand, the function of proteins and peptides are annotated in a systematic fashion through the Gene Ontology consortium [15]; in such effort, three aspects are recognized about the peptide function: molecular function (activity performed by the peptide without considering where or in what context the action takes place), cellular component (the cellular location where the peptide resides) and biological process (the cellular program accomplished by multiple activities). It is recognizable that since both peptide structure and peptide function are observed from the same molecule, these two observations should be related. However, it is not clear so far what is the form of such relationship. Recognizing the current limitation to relate protein structure and function, an international experiment has been conducted in the past decade to improve the formalization of the structure–function relationship of proteins; the experiment is referred to as the Critical Assessment of Function Annotation algorithms (CAFA) and it is currently in its 4th edition (CAFA4) [16]. The most recent report from CAFA3 showed that simple sequence comparison does not improve machine-learning (ML) methods to relate structure and function in proteins, and that the cellular localization (F_max_ < 0.7), molecular function (F_max_ < 0.7), and biological process (F_max_ < 0.5) are predicted in that order by the best ML methods; furthermore, small improvements have been observed in the performance of these methods despite the increasing number of data included by the methods, suggesting that the current descriptors/methods used for that goal are improvable [17].

The annotation of peptides as AMP refers to the molecular function according to the gene ontology consortium (see above). AMP are produced by unicellular and multicellular organisms [18]; they constitute a line of defense against other competing organisms such as bacteria, fungi, or viruses. But these peptides also have the capacity to regulate the immune system [19], control angiogenesis, and wound healing [20] as well as prevent tumor growth [21]; such functions correspond to biological processes. We may anticipate then, that AMP should be better predicted than angiogenesis or immunomodulation based on the CAFA experience, unless these biological processes are associated by a common mechanism and consequently may be related by common structural/chemical descriptors.

In that sense, the reported mechanisms triggered by AMP associated with these activities are diverse, yet some similarities may be recognized. For instance, AMP may kill bacteria by creating membrane pores [22]. This mechanism of action has been reported for cationic and amphipathic AMP interacting with bacterial membranes, which possess many negatively charged groups on their membranes. This mechanism has been reported also relevant to explain the antitumor activity of AMP, since in these cells the negatively charged phosphatidylserine, heparan sulfate, O-glycosylated mucins, and sialylated gangliosides are exposed to the outside of cell membrane; this membrane composition in combination with an increased transmembrane potential and membrane fluidity makes these cells susceptible to the pore-forming mechanism of the positively charged AMP [23].

On the other hand, regulation of immune system and wound healing by AMP is accomplished by different mechanisms than the membrane pore-forming mechanism. AMP are known to bind chemokine receptors and through these induce pro- or anti-inflammatory effects [19]. The ability of AMP to bind other targets than membranes has been recognized as an alternative way to kill bacterial cells; for instance, AMP are known to bind ATP [24,25], DNA [26,27], and lipid II [28]. In all these cases, the AMP-interacting molecule is negatively charged; hence, it is possible that positively charged AMP may bind to proteins that possess a negatively charged surface. Indeed, several AMP displaying antiviral activity were reported to bind glycoproteins and such binding depended on the abundance of basic amino acids [29]. Whether such pattern is also observed in AMP–chemokine receptor interactions requires experimental evidence not currently available [30].

If the antimicrobial activity of AMP depends on interacting with DNA for instance, AMP should be able to penetrate cells. In fact, accumulating evidence shows that many AMP exert their antimicrobial activity by interacting with intracellular targets. For instance, several AMP are known to inhibit protein synthesis by interacting with the ribosome [31,32] or by regulating metabolic enzymes [33,34], among other targets [35,36].

Since AMP have multiple activities *in vivo*, these peptides should balance their ability to penetrate cells with their ability to interact with different targets either outside cells to form pores or inside cells, to ultimately activate different biological processes. It is expected then that different AMP may have tuned their multiple functions to act specifically in different cellular contexts. What are the structural features of peptides and how these are related to different AMP functions represents an important aspect of the structure–function relationship models conducted to predict AMP activity and a fundamental problem in molecular biology for many other polymers such as proteins, among others, as we will describe next. Of particular interest to our review is the relationship between cell-penetrating activity with antimicrobial activity.

## Modeling AMP activity from peptide structure

As noted above, there is an overlap between the different activities displayed by AMP that are associated with some structural properties of these peptides, particularly the net charge of peptides. But a single structural feature is unlikely to efficiently classify AMP from non-AMP and from the other activities displayed by these peptides. Here, we will review the most efficient methods to classify AMP and the structural features that more strongly relate with such activity. Later on, we will perform a similar analysis with CPP with the interest to compare AMP and CPP structural features.

Predictors of different AMPs’ activities are based on two main ML approaches: shallow- and deep-learning models. Shallow-learning models [37–44] require computing a set of structural descriptors to represent each peptide. The best up to date set of predictors are based on the Random Forest (RF) method [45]. The success of the method is based on the dataset used and on the modeling approach. The dataset comprises the largest collection of AMPs experimentally validated: 22642 peptide sequences from the StarpedDB database [46]. The training dataset is selected after a clustering process and by sampling, at random, 80% of the sequences in each resulting cluster for training and 20% for testing. The same selection procedure is followed for the positive (true AMP) and negative (non-AMP) instances.

To construct their set of non-AMP sequences, the authors in [45] downloaded from the UniProt database (v2019_08) [47] a total of 561046 peptides with no evidence of antimicrobial activity through the next two queries: (Golgi OR cytoplasm OR ‘endoplasmic reticulum’ OR mitochondria) AND NOT antimicrobial AND length: (5–100), and NOT antimicrobial AND reviewed: YES. Peptides with non-natural amino acids were excluded. By using the CD-HIT program [48], sequences are sorted by length and then alphabetically; these sorted sequences are grouped by percentage of identity (50% or more). The sequences are compared with the first sequence in the sorted list (the cluster’s representative), once the identity falls below 50%, a new cluster is created, the process is repeated until all sequences are considered. Finally, from the remaining set of non-AMPs, several peptide fragments were randomly selected to be part of the antibacterial, antifungal, antiparasitic, and antiviral sets with a one to one ratio for the positive (AMP)–negative (non-AMP) sequences. Similar queries have been used in other works to construct non-AMP sequences derived from UniProt [38,40,47,49]. The two queries used in [45] were the same as those proposed by Gabere and Noble [38] to carry out their benchmarking study on AMP binary classifiers.

The modeling part uses an ensemble of feature subsets derived from six feature selection algorithms. From the set of resulting features, a second round of feature selection is performed by using a Wrapper method [50] based on RF [51] as induction algorithm and a genetic algorithm (GA) as optimizer, the fitness function for the GA is the classification accuracy, the wrapper is implemented in the Weka framework [52].

In general, ML is based on the assumption that for a given learning target A, there is a function g(s) such that:

g:S−>A

where g(s) is a mathematical function from the peptide structure, s ϵ S, that is related to the biological function (the label of s) of the peptide, a ϵ A. Here, S is the universe of peptides and A is the set of biological functions. Thus, given a set of examples, S’ subset of S, an ML method will try to learn a function f(s) that is an approximation to g(s); the function f(s) uses the set of structural features described above. In consequence, the success of any ML method relies on a careful and systematic selection of the training dataset, followed by an optimal selection of structural features for each activity to be modeled. A relevance analysis of the structural features of AMP revealed that some of them, computed by the ProtDcal software [53], are repeatedly selected within the top five to discriminate AMPs, antibacterial, antifungal, antiviral, and antiparasitic activities. These structural descriptors are of the physicochemical type and include indices of heat of formation (DHf) and isotropic surface area (ISA), as well as the index of the interfacial free energy of an unfolded state [Gs(U)]. Deep-learning models [54–58] produce competitive results; however, they do not shed light on what the relevant descriptors are, hence, we will not attempt to make any sense of the structural features used by those methods in the present review. Notice, however, that there is a research area aimed at explaining what are the features the deep network recognizes for discriminating the classes, the field is known as eXplainable IA (XIA) [59].

## CPP structure–activity relationship

CPP annotation corresponds to a molecular function according to the gene ontology consortium (see above); hence, its classification may be efficiently learned by current function annotation methods (see below). CPP are peptides capable of internalizing into different cell types, and these are found both in nature or are purposely designed. Among the first CPP described was the homeodomain of Antennapedia, a 60-amino acid residue peptide with the ability to bind DNA that was later discovered to translocate across neuronal membranes to reach the nuclei of cells [60]; as noted above, AMP are also able to bind DNA presumably because of charge complementation. With the accumulation of sequences with AMP and CPP activities, it was noticed that there were structural similarities between AMP and CPP, and such similarities were imprinted in their sequences, that is, many AMP and CPP are cationic and amphipathic and some CPP display AMP activity and vice versa [61]. Such bivalent activity was shown to depend on the target rather than on the peptide [62]; hence, it is expected that some CPP may have this dual activity when the targets (microbes) are close by. Thus, the molecular function of CPP shares structural similarities with AMP as well as functional ones: some CPP and AMP are able to penetrate cell membranes, and some are able to bind DNA and display CPP or AMP activities; yet, these two classes of peptides are presumably not identical. This premise needs further testing, as we will summarize next.

CPP are currently studied as vectors to deliver molecules inside cells [63]; such deliverable molecules may be small drugs [64], siRNA [65], AMP [66], or probes to diagnose a disease [67]. CPP may penetrate cells through different mechanisms, namely: (i) endocytosis-mediated mechanism [68] or (ii) energy-independent mechanisms [69]. Both of these mechanisms are also found in AMP. For instance, it has been noted that the AMP named CGA-N12 kills fungal cells by internalizing cells using an energy-dependent mechanism via endocytosis [70]; alternatively, PAF26 is able to kill fungal cells at low concentrations using an energy-dependent mechanism via endocytosis, and at high fungicidal concentrations internalizes through an energy-independent mechanism [71]. But a combination of both mechanisms may also take place in some cases. For instance, if the CPP include a peptide sequence recognized by a receptor, the peptide may be internalized through receptor-mediated endocytosis [72], yet sometimes the internalization although may use the receptor, does not necessarily involve endocytosis [73]. Additionally, since some AMP are able to bind to external targets (e.g., receptors) in addition to their ability to penetrate cells, this dual functionality may as well be present among CPP. To test this idea, cells expressing the reported receptors known to bind to AMP should be analyzed. To the best of our knowledge, there are no CPP-receptor reported interactions, although there are many reports fusing CPP to peptide sequences recognized by receptors [72,74]. This fundamental question about the mechanism of action of CPP is against the notion that CPP are only able to translocate into cells.

Indeed, while initially CPP were considered innocuous to living cells [75], recently it has been recognized that some CPP induce autophagy [76]. This is an activity also shared with some AMP [77,78]. It has been proposed that the antibacterial activity and autophagy induction of AMP is the consequence of their ATP-binding activity [25], which may as well apply to CPP considering the cationic character of both AMP and CPP. Understanding which targets of AMP are relevant for their toxic activity will be relevant to differentiate AMP from CPP, and consequently to improve on the current designs of AMP and CPP.

## Modeling CPP activity from peptide structure

The CPP predictors can be classified as direct or phenomenological and ML-based approaches. In the first category, the methods compute physicochemical characteristics that are known to be related to the cell-penetration property of peptides, for instance, Z-descriptors, number of heavy atoms, among others. Here, a peptide is labeled as CPP if the computed physicochemical characteristics fall within some specified intervals computed from a set of experimentally validated CPP. Examples in this group have been reviewed elsewhere [79,80]. In the second category, the methods follow a standard ML approach, where a set of features are calculated, then a feature selection algorithm is applied, afterward an induction algorithm is trained based on a set of positive and negative samples. In this category, Dobchev et al. [81] were the first to propose an artificial neural network (ANN), followed by Sanders et al. [82] that proposed a support vector machine (SVM) model, years later Diener et al. [26] used SVM and RF models. The three methods explored different set of features, based on physicochemical descriptors. Besides, the first two approaches also included a feature selection component. In all of them, the physicochemical properties are the input features for the ML model. Many other ML-based approaches have been proposed [83–89] since the pioneer work of Dobchev et al. Central to the success of ML models is the quality and the size of the datasets. In this regard, the largest database is CPPsite 2.0 [90] with 1699 unique peptides, which is still a small number considering, for instance, the size of databases of other bioactive peptides that include more than 20000 sequences [46]. All approaches use experimentally validated CPP as their positive cases. However, the construction of the negative instances is not unique, some use random sequences, other use bioactive peptides that are not CPP, a few cases use experimentally validated non-CPP, yet there are only 34 validated non-CPP [86]. Notice that the construction of negative examples for CPP differs from the construction of negative samples for AMP.

Considering the diversity of CPP predictors, it is relevant to ask, which of all these methods is the best to identify true CPP? To partially answer this question, a comparison of web-based tools is provided by Su et al. [91]. According to their study, the server KELM-CPPpred in its variant KELM-hybrid-AAC achieved the highest performance measured by the Mathews Correlation Coefficient or MCC. The method uses a kernelized version of the extreme learning machine [92]. To the best of our knowledge, there are no deep-learning-based approaches for the binary classification of CPP, this can be explained by the small amount of available CPP to date that will allow shallow approaches to outperform deep models, as it has been recently shown to be the case for the classification of AMP [93].

A recent work [94] proposed a CPP classification model based on an ensemble of ANN, SVM, and a Gaussian Process Classifier. The results achieved by this ensemble outperform other state-of-the-art models such as MLCPP [95], CPPred-RF [96], and SkipCPP-Pred. The study also reveals that a combination of sequence- and structure-based descriptors along with physicochemical descriptors achieves competitive accuracy levels and requires a smaller number of descriptors (43) than those used when using only sequence-based or only structure-based descriptors. The most relevant descriptors according to the normalized cumulative information entropy are the pseudo amino acid compositions (PseAACs) and the structure-based features. Regarding the sequence-based features, the abundance of cationic residues, such as lysine and arginine, plays an important role. An optimal selected combination of features consists of structure-based and physicochemical features such as molecular weight (MW), 1-octanol/water partition coefficient (cLogP), the fraction of SP^3^-hybridized carbon atoms (Fsp3), hydrogen bond acceptors (HBA), number of aromatic rings (NAR), primary amine groups (NPA), number of guanidine groups (NG), net charge (NetC), number of negatively charged amino acid groups (NNCAA) (nine out of 12), sequence based such as fraction of Lysine residues (f[Lys]), fraction of Arginine residues (f[Arg]) (two out of 20 AACs), ten out of 40 dipeptide composition, and 22 out of 22 PseACC descriptors (see Table 4 in [94]). Other works also report MW and NetC as relevant for classifying antimicrobial activity [97,98]. Also, Fsp3 has recently being proposed as a drug-likeness measure [99]. However, further analysis is required to understand the relationship between the descriptors selected to discriminate CPPs from non-CPPs and those selected to discriminate AMPs from non-AMPs. Such an analysis might shed light about the mechanism of action of certain AMPs that permeate the membrane and interact with some internal cellular process. For that end, further experimental characterization of both AMP and CPP is also required.

Thus, it has been early recognized that AMP and CPP being cationic and amphipathic may share the ability to bind anions, such as ATP, and consequently may induce autophagy as well as control the growth of microbial communities. However, structure–function studies of AMP and CPP are discovering new physicochemical features that are relevant for these activities; it is interesting to note the lack of overlap between the AMP and CPP most relevant features that help classifying AMP from non-AMP and CPP from non-CPP. Thus, designing AMP with CPP activity may find some redundancy in terms of the cationic and amphipathic character, but other attributes should be considered to provide other functionalities such as immunomodulation or the ability to interact with other intracellular targets (see below). In the next two sections, we will summarize the relevance of autophagy and microbial communities for health, to justify the potential uses of designing AMP with CPP activity to health.

## Autophagy and animal health

Autophagy is a term used to describe the self-eating molecular process originally characterized in yeast cells [100]. In such process, the degradation is performed by the lysosomes (or vacuole in the case of yeast cells). Three types of autophagy have been identified [101]: (i) microautophagy, (ii) macroautophagy, and (iii) chaperone-mediated autophagy. These three types of autophagy correspond to different mechanisms involved in delivering cargo to the lysosome. Macroautophagy is the first and most commonly identified form of autophagy; it uses autophagosomes to engulf cargo and deliver it to the lysosome. Microautophagy does not involve autophagosomes, instead the lysosome directly engulfs the cargo. The chaperone-mediated autophagy uses chaperones to translocate proteins, DNA and RNA to the lysosome.

Due to the catabolic nature of autophagy, this process is relevant for multiple biological processes, such as immunity, early development, ageing, adaptive metabolism, among others; but autophagy not only degrades the self-contents of cells, it also eliminates invading pathogens, paternal mitochondria, and others [102]. Such degradation process provides then the substrate to synthesize new cellular components, thus it is a fundamental component of allostasis and/or homeostasis.

Consequently, it has been observed that genetic mutations affecting autophagic genes promote cellular degeneration [103,104], promote early-age-related phenotypes [105,106], tumor development [107,108], and susceptibility to infections in animals [109,110]. Similarly, mutations of genes related to autophagy are present in several Mendelian diseases in humans such as Rett’s syndrome, Parkinson’s disease, cataracts, several forms of cardiomyopathies, among others [111].

The focus of the present review is on the relationship between autophagy, bacterial infections, and AMP/CPP. In that sense, it is important to define that an infection is considered any overgrowth of a microorganism within the host that affects its health. Microorganisms that affect health are considered pathogens [112], yet some nonpathogenic microorganisms cause disease states in immunocompromised patients or would infect patients with an underlying disease [113]; hence, the term pathogen is not always precise [114]. Furthermore, bacterial infections are found either inside host cells (e.g., human cells) or outside of them; such condition may differentiate the use of autophagy to deal with the invader, that is, only internal bacterial infections are subjected to xenophagy, the autophagic mechanism to deal with cellular invaders [115,116], which are recognized by macroautophagy [117]. This observation emphasizes the relevance of having AMP with CPP activity: these AMP will exert their activity by activating autophagy as well as by acting directly against the microbes, among other activities of relevance (e.g., immunomodulation).

When microbes are internalized in host cells and cause infections, the invaded cells activate autophagy; hence, the invading microbes must inhibit that response to survive. When invaded cells activate autophay, this also down-regulates the inflammation response [118,119]. Thus, if autophagy is inhibited by the invading microbe, then inflammation is activated [120] and may eliminate bacteria that are outside of cells. In such scenario, only bacteria residing inside cells will survive. Alternatively, if autophagy is not inhibited, inflammation will resume and bacteria residing inside will be eliminated while those outside of cells may be eliminated by the primary immune response, which includes AMP [121]. In the first case when intruder microorganisms are able to inhibit autophagy, these microbes may be eliminated by further activating autophagy [122,123]; in agreement with these observations, it has been reported that autophagy induction may prevent infections and other diseases [124]. Recognizing that AMP and CPP may induce autophagy it was proposed that AMP may have the dual ability to kill infectious bacteria through direct antibacterial activity and through autophagy [125], and now there is experimental evidence both in cells and in animals that such hypothesis is true [25,77].

Thus, AMP are a natural source of compounds that activate the immune system, activate autophagy, and have direct antimicrobial activity. Such combination of activities has been shown to be useful in treating diseases such as specific viral and bacterial infections. In the next section, we explore evidence that AMP may be used as well to maintain health; hence, these may as well be used to prevent diseases (prophylactic), once we have a better understanding on how to design them.

## AMP as prophylactic natural compounds to control microbiome establishment and animal health

Microbiota is the term used to refer to the microorganisms that live together with a multicellular host (e.g., humans, mice), that in combination with host cells provide the host with increased capabilities [126–129]. Nowadays, it is recognized that the multiple roles such microbiota play in animal health; for instance, the microbiota residing in the gut is relevant for the brain–gut axis, which in turn is relevant for behavior and the etiology of neuropsychiatric and neurodegenerative disorders [130], the gut microbiota is relevant for the immune system development [131] or the host metabolism performance [132,133], among others [127].

Thus, once the microbiota is established, this community assists the host in multiple tasks. But how is the microbiota established? That process may require the adaptability of host immune response to control the size of the different microbes populations. In that sense, it has been recognized the relevance of the mother in the newborn initial microbiota colonization [134,135]. Most of the microbiota from the mother may have the possibility to be transmitted to the newborn after birth [136]. During pregnancy, the mother predominantly uses the T-helper 2-type immunity (anti-inflammatory), to not affect the development of the fetus, hence promoting such immunity in the fetus immune system as well. But this immunity type gradually changes as the baby is ready to be born and during the first weeks of life it turns into Th1-type activity (proinflammatory). At birth, the newborn is exposed to the microbiota from the mother through the newborn skin; it has been reported that both mice and human newborns overexpress AMP in their skin [137] as a way to control first microbial infections. Later on, during the first months of life, while the adaptive immune response is not established, the infants depend mostly on the innate immune system; AMP are an essential component of this system and the relevance of AMP for the healthy newborn survival has been recently reviewed [138]. Among the AMP that have been detected in newborns are cathelicidins; a well-studied cathelicidin is the LL-37 AMP shown to induce autophagy [139]. As noted above, autophagy plays an anti-inflammatory role that is important for the newborns to deal with the continuous exposition and acquisition of new microorganisms. AMP are known to up-/down-regulate the release of chemokines and cytokines [140]; chemokines and cytokines are known to exert direct antimicrobial activity [141], suggesting that AMP may enhance the antimicrobial arsenal or reduce it, through the regulation of expression of these cytokines. Furthermore, cytokines are also able to up-/down-regulate autophagy [142]; hence, AMP is part of a connected network of molecular events that regulates the innate immune system response.

Later in life, microbiota constantly change to adapt to multiple internal and external conditions. Such changes are accompanied by AMP production as well. Examples of this are also found in the gut microbiota in animals, where diverse studies have shown a relationship between gut microbiota composition and health in adults [143]; for instance, changes in the gut microbiome (the microbiota inferred from sequencing techniques) are associated with cardiovascular disease [144]. To control the microbiota, the mammalian immune system uses the inflammatory system; a component of this system is the inflammosome, a protein complex containing pattern-recognition receptors (e.g., NLRP3) that upon recognition of its target activates the inflammatory response. Such activation should be regulated, otherwise may lead to dysbiosis [145]. For instance, an activating mutation in the NLRP3 receptor has been associated with autoinflammatory conditions on the skin [146], but no apparent pathology on the intestines [147]. The absence of any pathological state in the intestines of animals carrying this activating NLRP3 mutation comes with an altered composition of the gut microbiota, which provides resistance to inflammation; furthermore, such altered composition of the microbiota is the result of producing AMP by IL-1β cytokine [148]. Many other examples have been documented where AMP help control the microbiota in animals [149]. Thus, AMP play a role on controlling microbiota early in life and during the rest of the life of hosts; consequently, AMP sequences may have been adapted during the evolution of the species [143]. In summary, AMP are involved in controlling microbiota establishment and composition during our life, hence acting as natural prophylactic drugs.

## AMP as therapeutic drugs

The aim of the present review is not to cover all different peptides clinically approved to treat infections in humans. Instead, we have analyzed the multiple functions that AMP and CPP have on cells and in whole animals. The relevance of such analysis is to highlight the multifunctional nature of such peptides that are relevant to health under natural conditions: control of autophagy and microbiota. AMP have been used in clinical trials to treat different microbial infections, yet the mechanism of action of such AMP is not based on the combination of autophagy, immune regulation, and direct antimicrobial activities. The last AMP approved to treat microbial infections was daptomycin back in 2003 [150], despite the fact that the motivation to use AMP is their multifunctional capacity and consequently, the reduced chance to promote microbial resistance.

A recent review about the last two decades of failure to approve AMP in clinical trials noted different limitations of these AMP [151]; among others: (i) AMP may be toxic to human cells, (ii) AMP may lose activity under physiological salt conditions, (iii) AMP in the presence of serum may bind to proteins such as albumin, and (iv) AMP are susceptible to protease degradation, among others that will reduce their bioavailability. Most of these limitations are being addressed by different strategies such as chemical modifications to prevent proteolysis, shorten the AMP to reduce their capacity to interact with other proteins or include these within delivery systems to target the action of AMP. In that same review, 43 AMP that are under clinical investigation were identified; among these 43 AMP only 14 are recognized as multifunctional, in this case to display a direct antimicrobial activity through membrane disruption and to display an immunomodulatory activity (see Table 1).

**Table 1:** Multifunctional AMP in clinical trials

Peptide name	Administration	Phase	Target	Structure
Gramicidin	Topical	III	Infected wounds and ulcers	Polycyclic lantibiotic
Polymixin B	Topical	III	Gram-negative bacteria	Cyclic polypeptide
Polymixin E (Colistin)	Intravenus	III	*A. baumannii*/Pneumonia	Cyclic polypeptide
Daptomycin	Intravenous	III	Skin infection/bacteremia	Lipopeptide
LL-37	Topical	II	Leg ulcers	Human cathelicidn
Melittin	Intradermal	I/II	Inflammation	Alpha-helical peptide
Pexiganan (MSI-78)	Topical	III	Diabetic foot ulcers	Alpha-helical peptide
Omiganan (MBI-226)	Topical	III	Antisepsis/catheter infections	Derivative of indolicidine
OP-145	Ear drops	I/II	Chronic middle ear infection	Derivative of LL-37
P113 (PAC-113)	Mouth rinse	II	Oral candidiasis	Fragment of histatin-5
hLF1-11	Intravenous	I/II	Bacterial/fungal infections	Fragment of human lactoferrin
DPK-060	Ear drops	II	Acute external otitis	Derivative of kininogen
AP-214	Intravenous	II	Postsurgical organ failure	Derivative of α-MSH
PMX-30063	Intravenous	II	Acute bacterial skin infection	Defensin mimetic

Five out of these 14 AMP are administered intravenously (Polymixin E, Daptomycin, hLK1-11, AP-214, and PMX-30063); hence, it is likely that their pharmaceutical action should overcome all the noted limitations of AMP above. It will be relevant to review these and other AMP that will enter human trials to learn how to continue developing new AMP that are able to control microbial infections in a complex biological system; of particular interest will be the molecular features of such AMP. For instance, are these AMP capable of inducing autophagy? Are these AMP also CPP? Which sequence/physicochemical/topological features differentiate these successful AMP from those that failed clinical trials? As more examples of successful multifunctional AMP with CPP activity are registered, such information may eventually be helpful in designing AMP to control gut microbiota.

## Conclusion

**Figure 1:**
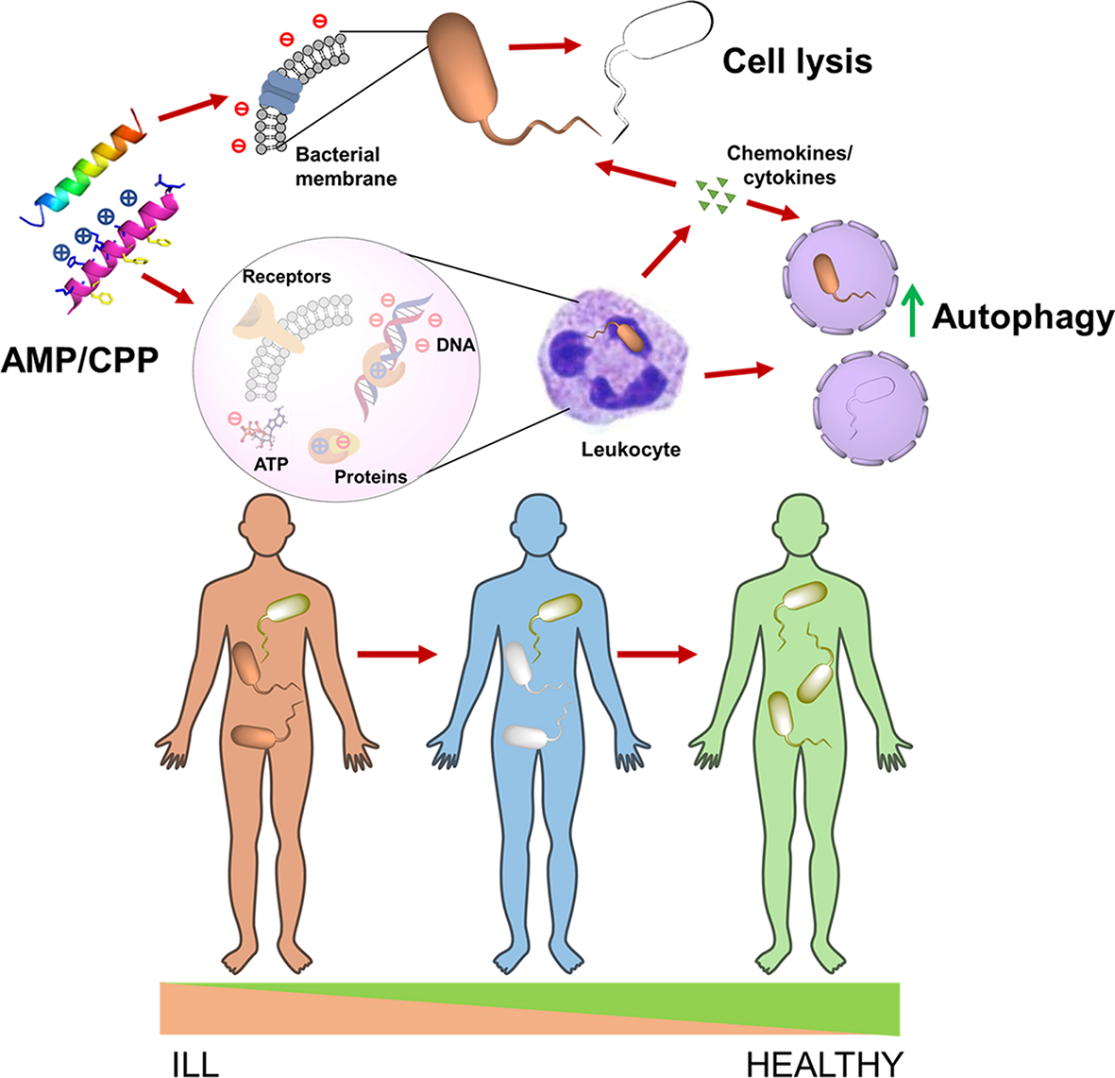
The relevance of multifunctional AMP for health AMP are amphipathic and cationic endowing interaction with anionic bacterial membranes, creating pores, and directly causing cell lysis. Furthermore, AMP with CPP activities also interact with cellular components such as DNA and ATP, which possess negatively charged groups, causing a disruption of their metabolism in bacteria. In some cell types like immune cells, AMP interact with receptors and/or induce autophagy, the release of cytokines and chemokines (with intrinsic antimicrobial activity), hence, promoting a feedback loop for autophagy resulting in the encapsulation and killing of bacteria (xenophagy). By considering these multiple functions of AMP with CPP activity, it may be possible to learn how the human body controls microbiota and autophagy to improve the health of the human population. Image created using ChemDraw.

Our review shows that AMP are multifunctional. Due to the cationic and amphipathic nature of AMP, these may bind anions such as DNA or ATP; for that end, AMP may need to penetrate cells and in doing so, AMP may activate autophagy. Furthermore, AMP may be recognized by receptors to regulate the production of cytokines, which may also act as antimicrobials directly or regulate the production of AMP. While these are generalities of many AMP, for this to act in a specific way, it is important to decipher the differences between penetrating from nonpenetrating AMP; for that end, CPP may provide some guidance. Furthermore, AMP and CPP may directly interact with proteins (e*.*g., receptors) that may also explain their multifunctionality. What are the structural properties of AMP that allow them bind selectively to different proteins remains unclear (see Figure 1).

## Abbreviations

AMP: antimicrobial peptides
ANN: artificial neural network
CAFA: critical assessment of function annotation
CPP: cell-penetrating peptides
FDA: Food and Drug Administration
Fsp3: fraction of SP3-hybridized carbon atoms
GA: genetic algorithm
HBA: hydrogen bond acceptor
ISA: isotropic surface area
MCC: Mathews correlation coefficient
ML: machine learning
MW: molecular weight
NAR: number of aromatic ring
NetC: net charge
NG: number of guanidine groups
NNCAA: number of negatively charged amino acid groups
NPA: Primary amine group
PseAAC: pseudo amino acid composition
RF: random forest
SVM: support vector machine
XIA: eXplainable Artificial Intelligence

## References

[1] Chen I. (2019) An antisense oligonucleotide splicing modulator to treat spinal muscular atrophy. Nat. Res. 2021, S17–S18, https://www.nature.com/articles/d42859-019-00090-4

[2] Fosgerau K. and Hoffmann T. (2015) Peptide therapeutics: current status and future directions. Drug Discov. Today 20, 122–128 10.1016/j.drudis.2014.10.003 25450771

[3] Scott D.E., Bayly A.R., Abell C. and Skidmore J. (2016) Small molecules, big targets: drug discovery faces the protein-protein interaction challenge. Nat. Rev. Drug Discov. 15, 533–550 10.1038/nrd.2016.29 27050677

[4] Villoutreix B., Bastard K., Sperandio O., Fahraeus R., Poyet J.-L. and Calvo F. (2008) In silico-in vitro screening of protein-protein interactions: towards the next generation of therapeutics. Curr. Pharm. Biotechnol. 9, 103–122 10.2174/138920108783955218 18393867

[5] Marr A.K., Gooderham W.J. and Hancock R.E. (2006) Antibacterial peptides for therapeutic use: obstacles and realistic outlook. Curr. Opin. Pharmacol. 6, 468–472 10.1016/j.coph.2006.04.006 16890021

[6] Otvos L. and Wade J.D. (2014) Current challenges in peptide-based drug discovery. Front. Chem. 2, 1–4 10.3389/fchem.2014.00062 PMC4126357 25152873

[7] Crews D.E. and Tuggle A.C. (2021) What is Health? Allostasis and the Evolution of Human Design (Sterling P., ed.), The MIT Press, 2020, Cambridge, MA, USA 10.1002/ajhb.23698 34779544

[8] Coste S.C. (2015) Allostasis, Homeostasis, and the Costs of Physiological Adaptation(Schulkin J., ed.), pp. 55–56, Cambridge University Press, Cambridge and New York, $100.00. xii + 372 p; ill.; index. ISBN: 0-521-81141-4. 2004 https://www.journals.uchicago.edu/doi/abs/10.1086/503934

[9] Dye C. (2021) The Great Health Dilemma: Is Prevention Better than Cure? - Christopher Dye - Google Libros, OUP, Oxford, https://books.google.com.mx/books?id=GxUuEAAAQBAJ&pg=PA1&hl=es&source=gbs_toc_r&cad=3#v=onepage&q&f=false

[10] Traniello J.F.A., Rosengaus R.B. and Savoie K. (2002) The development of immunity in a social insect: evidence for the group facilitation of disease resistance. Proc. Natl. Acad. Sci. U.S.A. 99, 6838–6842 10.1073/pnas.102176599 12011442 PMC124490

[11] Romero-Molina S., Ruiz-Blanco Y.B., Green J.R. and Sanchez-Garcia E. (2019) ProtDCal-Suite: a web server for the numerical codification and functional analysis of proteins. Protein Sci. 28, 1734–1743 10.1002/pro.3673 31271472 PMC6699089

[12] Liu B., Liu F., Wang X., Chen J., Fang L. and Chou K.C. (2015) Pse-in-One: a web server for generating various modes of pseudo components of DNA, RNA, and protein sequences. Nucleic Acids Res. 43, W65–W71 10.1093/nar/gkv458 25958395 PMC4489303

[13] Li Z.R., Lin H.H., Han L.Y., Jiang L., Chen X. and Chen Y.Z. (2006) PROFEAT: a web server for computing structural and physicochemical features of proteins and peptides from amino acid sequence. Nucleic Acids Res. 34, W32–W37 10.1093/nar/gkl305 16845018 PMC1538821

[14] Shen H.B. and Chou K.C. (2008) PseAAC: a flexible web server for generating various kinds of protein pseudo amino acid composition. Anal. Biochem. 373, 386–388 10.1016/j.ab.2007.10.012 17976365

[15] Gene Ontology Consortium (2021) The Gene Ontology resource: enriching a GOld mine. Nucleic Acids Res. 49, D325–D334 10.1093/nar/gkaa1113 33290552 PMC7779012

[16] The Function Special Interest Group. The CAFA challenge. https://www.biofunctionprediction.org/cafa/ Accessed on June 2022

[17] Zhou N., Jiang Y., Bergquist T.R., Lee A.J., Kacsoh B.Z., Crocker A.W. et al. (2019) The CAFA challenge reports improved protein function prediction and new functional annotations for hundreds of genes through experimental screens. Genome Biol. 20, 244 10.1186/s13059-019-1835-8 31744546 PMC6864930

[18] Huan Y., Kong Q., Mou H. and Yi H. (2020) Antimicrobial peptides: classification, design, application and research progress in multiple fields. Front. Microbiol. 11, 2559 10.3389/fmicb.2020.582779 PMC7596191 33178164

[19] Liang W. and Diana J. (2020) The dual role of antimicrobial peptides in autoimmunity. Front. Immunol. 11, 2077 10.3389/fimmu.2020.02077 32983158 PMC7492638

[20] Takahashi M., Umehara Y., Yue H., Trujillo-Paez J.V., Peng G. et al. (2021) The antimicrobial peptide human β-defensin-3 accelerates wound healing by promoting angiogenesis, cell migration, and proliferation through the FGFR/JAK2/STAT3 signaling pathway. Front. Immunol. 12, 3745 10.3389/fimmu.2021.712781 PMC8476922 34594328

[21] Roudi R., Syn N.L. and Roudbary M. (2017) Antimicrobial peptides as biologic and immunotherapeutic agents against cancer: a comprehensive overview. Front. Immunol. 8, 1–10 10.3389/fimmu.2017.01320 29081781 PMC5645638

[22] Lee T.-H., Hall K.N. and Aguilar M.-I. (2016) Antimicrobial peptide structure and mechanism of action: a focus on the role of membrane structure. Curr. Top. Med. Chem. 16, 25–39 10.2174/1568026615666150703121700 26139112

[23] Kumar P., Kizhakkedathu J.N. and Straus S.K. (2018) Antimicrobial peptides: diversity, mechanism of action and strategies to improve the activity and biocompatibility in vivo. Biomolecules 8, 25–39 https://pmc/articles/PMC5871973/ 10.3390/biom8010004 PMC5871973 29351202

[24] Hilpert K., McLeod B., Yu J., Elliott M.R., Rautenbach M. et al. (2010) Short cationic antimicrobial peptides interact with ATP. Antimicrob. Agents Chemother. 54, 4480 https://pmc/articles/PMC2944583/ 10.1128/AAC.01664-09 20660668 PMC2944583

[25] Coyotl E.A.P., Palacios J.B., Muciño G., Moreno-Blas D., Costas M. et al. (2020) Antimicrobial peptide against *Mycobacterium tuberculosis* that activates autophagy is an effective treatment for tuberculosis. Pharmaceutics 12, 1–24 10.3390/pharmaceutics12111071 PMC7697726 33182483

[26] Diener C., Garza Ramos Martínez G., Moreno Blas D., Castillo González D.A., Corzo G. et al. (2016) Effective design of multifunctional peptides by combining compatible functions. PLoS Comput. Biol. 12, 1–19 10.1371/journal.pcbi.1004786 27096600 PMC4838304

[27] Huo L., Zhang K., Ling J., Peng Z., Huang X. et al. (2011) Antimicrobial and DNA-binding activities of the peptide fragments of human lactoferrin and histatin 5 against *Streptococcus mutans*. Arch. Oral. Biol. 56, 869–876 10.1016/j.archoralbio.2011.02.004 21382611

[28] Malanovic N. and Lohner K. (2016) Antimicrobial peptides targeting Gram-positive bacteria. Pharmaceuticals (Basel) 9, 1–33 10.3390/ph9030059 27657092 PMC5039512

[29] Zhao H., Zhou J., Zhang K., Chu H., Liu D. et al. (2016) A novel peptide with potent and broad-spectrum antiviral activities against multiple respiratory viruses. Sci. Rep. 6, 1–13 10.1038/srep22008 26911565 PMC4766503

[30] Mahlapuu M., Håkansson J., Ringstad L. and Björn C. (2016) Antimicrobial peptides: an emerging category of therapeutic agents. Front. Cell Infect. Microbiol. 6, 194 10.3389/fcimb.2016.00194 28083516 PMC5186781

[31] Graf M., Mardirossian M., Nguyen F., Seefeldt A.C., Guichard G. et al. (2017) Proline-rich antimicrobial peptides targeting protein synthesis. Nat. Prod. Rep. 34, 702–711 10.1039/C7NP00020K 28537612

[32] Boman H.G., Agerberth B. and Boman A. (1993) Mechanisms of action on *Escherichia coli* of cecropin P1 and PR-39, two antibacterial peptides from pig intestine. Infect. Immun. 61, 2978–2984 10.1128/iai.61.7.2978-2984.1993 8514403 PMC280948

[33] Braffman N.R., Piscotta F.J., Hauver J., Campbell E.A., James Link A. et al. (2019) Structural mechanism of transcription inhibition by lasso peptides microcin J25 and capistruin. Proc. Natl. Acad. Sci. U.S.A. 116, 1273–1278 10.1073/pnas.1817352116 30626643 PMC6347699

[34] Kragol G., Lovas S., Varadi G., Condie B.A., Hoffmann R. et al. (2001) The antibacterial peptide pyrrhocoricin inhibits the ATPase actions of DnaK and prevents chaperone-assisted protein folding. Biochemistry 40, 3016–3026 10.1021/bi002656a 11258915

[35] Lehrer R.I., Barton A., Daher K.A., Harwig S.S.L., Ganz T. et al. (1989) Interaction of human defensins with *Escherichia coli*. Mechanism of bactericidal activity. J. Clin. Invest. 84, 553–561 10.1172/JCI114198 2668334 PMC548915

[36] Lv Y., Shao G., Zhang Q., Wang X., Meng Y. et al. (2019) The antimicrobial peptide PFR induces necroptosis mediated by ER stress and elevated cytoplasmic calcium and mitochondrial ROS levels: cooperation with Ara-C to act against acute myeloid leukemia. Signal Transduct. Target Ther. 4, 1–3 10.1038/s41392-019-0073-6 31637016 PMC6799817

[37] Schaduangrat N., Nantasenamat C., Prachayasittikul V. and Shoombuatong W. (2019) Meta-iAVP: a sequence-based meta-predictor for improving the prediction of antiviral peptides using effective feature representation. Int. J. Mol. Sci. 20, 1–25 10.3390/ijms20225743 PMC6888698 31731751

[38] Gabere M.N. and Noble W.S. (2017) Empirical comparison of web-based antimicrobial peptide prediction tools. Bioinformatics 33, 1921–1929 10.1093/bioinformatics/btx081 28203715 PMC5860510

[39] Joseph S., Karnik S., Nilawe P., Jayaraman V.K. and Idicula-Thomas S. (2012) ClassAMP: a prediction tool for classification of antimicrobial peptides. IEEE/ACM Trans. Comput. Biol. Bioinforma 9, 1535–1538 10.1109/TCBB.2012.89 22732690

[40] Xiao X., Wang P., Lin W.Z., Jia J.H. and Chou K.C. (2013) iAMP-2L: a two-level multi-label classifier for identifying antimicrobial peptides and their functional types. Anal. Biochem. 436, 168–177 10.1016/j.ab.2013.01.019 23395824

[41] Meher P.K., Sahu T.K., Saini V. and Rao A.R. (2017) Predicting antimicrobial peptides with improved accuracy by incorporating the compositional, physico-chemical and structural features into Chou's general PseAAC. Sci. Rep. 7, 1–12 10.1038/srep42362 28205576 PMC5304217

[42] Gupta S., Kapoor P., Chaudhary K., Gautam A., Kumar R. et al. (2013) In silico approach for predicting toxicity of peptides and proteins. PloS ONE 8, 1–10 10.1371/journal.pone.0073957 PMC3772798 24058508

[43] Bhadra P., Yan J., Li J., Fong S. and Siu S.W.I. (2018) AmPEP: Sequence-based prediction of antimicrobial peptides using distribution patterns of amino acid properties and random forest. Sci. Rep. 8, 1–10 10.1038/s41598-018-19752-w 29374199 PMC5785966

[44] Chung C.R., Kuo T.R., Wu L.C., Lee T.Y. and Horng J.T. (2019) Characterization and identification of antimicrobial peptides with different functional activities. Brief. Bioinform. 21, 1098–1114 10.1093/bib/bbz043 31155657

[45] Pinacho-Castellanos S.A., García-Jacas C.R., Gilson M.K. and Brizuela C.A. (2021) Alignment-free antimicrobial peptide predictors: improving performance by a thorough analysis of the largest available data set. J. Chem. Inf. Model. 61, 3141–3157 10.1021/acs.jcim.1c00251 34081438

[46] Aguilera-Mendoza L., Marrero-Ponce Y., Beltran J.A., Tellez Ibarra R., Guillen-Ramirez H.A. and Brizuela C.A. (2019) Graph-based data integration from bioactive peptide databases of pharmaceutical interest: toward an organized collection enabling visual network analysis. Bioinformatics 35, 4739–4747 10.1093/bioinformatics/btz260 30994884

[47] UniProt Consortium (2019) UniProt: a worldwide hub of protein knowledge. Nucleic Acids Res. 47, D506–D515 10.1093/nar/gky1049 30395287 PMC6323992

[48] Huang Y., Niu B., Gao Y., Fu L. and Li W. (2010) CD-HIT Suite: a web server for clustering and comparing biological sequences. Bioinformatics 26, 680–682 10.1093/bioinformatics/btq003 20053844 PMC2828112

[49] Torrent M., Andreu D., Nogués V.M. and Boix E. (2011) Connecting peptide physicochemical and antimicrobial properties by a rational prediction model. PloS ONE 6, e16968 10.1371/journal.pone.0016968 21347392 PMC3036733

[50] Kohavi R. and John G.H. (1997) Wrappers for feature subset selection. Artif. Intell. 97, 273–324 10.1016/S0004-3702(97)00043-X

[51] Breiman L. (2001) Random forest. Machine Learning 45, 5–32 10.1023/A:1010933404324

[52] Frank E., Hall M.A. and Witten I.H. WEKA Software. https://www.cs.waikato.ac.nz/ml/weka/index.html Accessed on June 2022

[53] Romero-Molina S., Ruiz-Blanco Y.B., Green J.R. and Sanchez-Garcia E. (2019) ProtDCal-Suite: a web server for the numerical codification and functional analysis of proteins. Protein Sci. 28, 1734–1743 10.1002/pro.3673 31271472 PMC6699089

[54] Veltri D., Kamath U. and Shehu A. (2018) Deep learning improves antimicrobial peptide recognition. Bioinformatics 34, 2740–2747 10.1093/bioinformatics/bty179 29590297 PMC6084614

[55] Su X., Xu J., Yin Y., Quan X. and Zhang H. (2019) Antimicrobial peptide identification using multi-scale convolutional network. BMC Bioinformatics 20, 1–10 10.1186/s12859-019-3327-y 31870282 PMC6929291

[56] Yan J., Bhadra P., Li A., Sethiya P., Qin L. et al. (2020) Deep-AmPEP30: improve short antimicrobial peptides prediction with deep learning. Mol. Ther. Nucleic Acids 20, 882–894 10.1016/j.omtn.2020.05.006 32464552 PMC7256447

[57] Hamid M.N. and Friedberg I. (2019) Identifying antimicrobial peptides using word embedding with deep recurrent neural networks. Bioinformatics 35, 2009–2016 10.1093/bioinformatics/bty937 30418485 PMC6581433

[58] Youmans M., Spainhour J.C.G. and Qiu P. (2020) Classification of antibacterial peptides using long short-term memory recurrent neural networks. IEEE/ACM Trans. Comput. Biol. Bioinforma 17, 1134–1140 10.1109/TCBB.2019.2903800 30843849

[59] Tjoa E. and Guan C. (2021) A survey on explainable artificial intelligence (XAI): toward medical XAI. IEEE Transact. Neural Networks Learning Systems 32, 4793–4813 10.1109/TNNLS.2020.3027314 33079674

[60] Derossit D., Joliott A.H., Chassaing G. and Prochiantz A. (1994) The third helix of the Antennapedia homeodomain translocates through biological membranes. J. Biol. Chem. 269, 10444–10450 10.1016/S0021-9258(17)34080-2 8144628

[61] Zorko M. and Langel Ü. (2005) Cell-penetrating peptides: mechanism and kinetics of cargo delivery. Adv. Drug. Deliv. Rev. 57, 529–545 10.1016/j.addr.2004.10.010 15722162

[62] Rodriguez Plaza J.G., Morales-Nava R., Diener C., Schreiber G., Gonzalez Z.D. et al. (2014) Cell penetrating peptides and cationic antibacterial peptides: two sides of the same coin. J. Biol. Chem. 289, 14448–14457 10.1074/jbc.M113.515023 24706763 PMC4031501

[63] Heitz F., Morris M.C. and Divita G. (2009) Twenty years of cell-penetrating peptides: from molecular mechanisms to therapeutics. Br. J. Pharmacol. 157, 195–206 10.1111/j.1476-5381.2009.00057.x 19309362 PMC2697800

[64] Bates E., Bode C., Costa M., Gibson C.M., Granger C. et al. (2008) Intracoronary KAI-9803 as an adjunct to primary percutaneous coronary intervention for acute ST-segment elevation myocardial infarction. Circulation 117, 886–896 10.1161/CIRCULATIONAHA.107.759167 18250271

[65] Eguchi A. and Dowdy S.F. (2009) siRNA delivery using peptide transduction domains. Trends Pharmacol. Sci. 30, 341–345 10.1016/j.tips.2009.04.009 19545914

[66] Rodríguez Plaza J.G., Villalón Rojas A., Herrera S., Garza-Ramos G., Torres Larios A. et al. (2012) Moonlighting peptides with emerging function. PloS ONE 7, e40125 https://journals.plos.org/plosone/article?id=10.1371/journal.pone.0040125 10.1371/journal.pone.0040125 22808104 PMC3396687

[67] Tripathi P.P., Arami H., Banga I., Gupta J. and Gandhi S. (2018) Cell penetrating peptides in preclinical and clinical cancer diagnosis and therapy. Oncotarget 9, 37252–37267 10.18632/oncotarget.26442 30647857 PMC6324683

[68] Richard J.P., Melikov K., Vives E., Ramos C., Verbeure B. et al. (2003) Cell-penetrating peptides. A re-evaluation of the mechanism of cellular uptake. J. Biol. Chem. 278, 585–590 10.1074/jbc.M209548200 12411431

[69] Patel L.N., Zaro J.L. and Shen W.C. (2007) Cell penetrating peptides: intracellular pathways and pharmaceutical perspectives. Pharm. Res. 24, 1977–1992 10.1007/s11095-007-9303-7 17443399

[70] Li R., Tao M., Li S., Wang X., Yang Y., Mo L. et al. (2021) Internalization and membrane activity of the antimicrobial peptide CGA-N12. Biochem. J. 478, 1907–1919 10.1042/BCJ20201006 33955460

[71] Muñoz A., Marcos J.F. and Read N.D. (2012) Concentration-dependent mechanisms of cell penetration and killing by the de novo designed antifungal hexapeptide PAF26. Mol. Microbiol. 85, 89–106 10.1111/j.1365-2958.2012.08091.x 22646057

[72] Morán-Torres R., Castillo González D.A., Durán-Pastén M.L., Aguilar-Maldonado B., Castro-Obregón S. et al. (2021) Selective moonlighting cell-penetrating peptides. Pharmaceutics 13, 10.3390/pharmaceutics13081119 34452080 PMC8400200

[73] Del Rio G., Klipp E. and Herrmann A. (2017) Using confocal microscopy and computational modeling to investigate the cell-penetrating properties of antimicrobial peptides. Methods Mol. Biol. 1548, 191–199 10.1007/978-1-4939-6737-7_13 28013505

[74] Jung S.W., Seo J.W., Park S.H., Kim Y.G., Moon J.Y., Choi S. et al. (2021) A cell-penetrating peptide that blocks toll-like receptor signaling protects kidneys against ischemia-reperfusion injury. Int. J. Mol. Sci. 22, 1627 10.3390/ijms22041627 33562802 PMC7915942

[75] Suhorutsenko J., Oskolkov N., Arukuusk P., Kurrikoff K., Eriste E. et al. (2011) Cell-penetrating peptides, PepFects, show no evidence of toxicity and immunogenicity in vitro and in vivo. Bioconjug. Chem. 22, 2255–2262 10.1021/bc200293d 21978260

[76] Dowaidar M., Gestin M., Cerrato C.P., Jafferali M.H., Margus H. et al. (2017) Role of autophagy in cell-penetrating peptide transfection model. Sci. Rep. 7, 1–14 10.1038/s41598-017-12747-z 28974718 PMC5626743

[77] Sultana Rekha R., Rao Muvva S.J., Wan M., Raqib R., Bergman P. et al. (2015) Phenylbutyrate induces LL-37-dependent autophagy and intracellular killing of *Mycobacterium tuberculosis* in human macrophages. Autophagy 11, 1688–1699 10.1080/15548627.2015.1075110 26218841 PMC4590658

[78] Bera A., Singh S., Nagaraj R. and Vaidya T. (2003) Induction of autophagic cell death in *Leishmania donovani* by antimicrobial peptides. Mol. Biochem. Parasitol. 127, 23–35 10.1016/S0166-6851(02)00300-6 12615333

[79] Hällbrink M. and Karelson M. (2015) Prediction of cell-penetrating peptides. Methods Mol. Biol. 1324, 39–58 10.1007/978-1-4939-2806-4_3 26202261

[80] Hansen M., Kilk K. and Langel U. (2008) Predicting cell-penetrating peptides. Adv. Drug. Deliv. Rev. 60, 572–579 10.1016/j.addr.2007.09.003 18045726

[81] Dobchev D.A., Mager I., Tulp I., Karelson G., Tamm T., Tamm K. et al. (2010) Prediction of cell-penetrating peptides using artificial neural networks. Curr. Comput. Aided Drug. Des. 6, 79–89 10.2174/157340910791202478 20402661

[82] Sanders W.S., Johnston C.I., Bridges S.M., Burgess S.C. and Willeford K.O. (2011) Prediction of cell penetrating peptides by support vector machines. PLoS Comput. Biol. 7, e1002101 10.1371/journal.pcbi.1002101 21779156 PMC3136433

[83] Wei L., Xing P., Su R., Shi G., Ma Z.S. et al. (2017) CPPred-RF: a sequence-based predictor for identifying cell-penetrating peptides and their uptake efficiency. J. Proteome Res. 16, 2044–2053 10.1021/acs.jproteome.7b00019 28436664

[84] Wei L., Tang J. and Zou Q. (2017) SkipCPP-Pred: an improved and promising sequence-based predictor for predicting cell-penetrating peptides. BMC Genomics 18, 1–11 10.1186/s12864-017-4128-1 29513192 PMC5657092

[85] Holton T.A., Pollastri G., Shields D.C. and Mooney C. (2013) CPPpred: prediction of cell penetrating peptides. Bioinformatics 29, 3094–3096 10.1093/bioinformatics/btt518 24064418

[86] Chen L., Chu C., Huang T., Kong X. and Cai Y.D. (2015) Prediction and analysis of cell-penetrating peptides using pseudo-amino acid composition and random forest models. Amino Acids 47, 1485–1493 10.1007/s00726-015-1974-5 25894890

[87] Tang H., Su Z.D., Wei H.H., Chen W. and Lin H. (2016) Prediction of cell-penetrating peptides with feature selection techniques. Biochem. Biophys. Res. Commun. 477, 150–154 10.1016/j.bbrc.2016.06.035 27291150

[88] Pandey P., Patel V., George N.V. and Mallajosyula S.S. (2018) KELM-CPPpred: Kernel extreme learning machine based prediction model for cell-penetrating peptides. J. Proteome Res. 17, 3214–3222 10.1021/acs.jproteome.8b00322 30032609

[89] Qiang X., Zhou C., Ye X., Du P.F., Su R. and Wei L. (2018) CPPred-FL: a sequence-based predictor for large-scale identification of cell-penetrating peptides by feature representation learning. Brief. Bioinform. 21, 11–23 10.1093/bib/bby091 30239616

[90] Agrawal P., Bhalla S., Usmani S.S., Singh S., Chaudhary K., Raghava G.P. et al. (2016) CPPsite 2.0: a repository of experimentally validated cell-penetrating peptides. Nucleic Acids Res. 44, D1098–D1103 10.1093/nar/gkv1266 26586798 PMC4702894

[91] Su R., Hu J., Zou Q., Manavalan B. and Wei L. (2020) Empirical comparison and analysis of web-based cell-penetrating peptide prediction tools. Brief. Bioinform. 21, 408–420 10.1093/bib/bby124 30649170

[92] Huang G.B. (2015) What are extreme learning machines? Filling the gap between Frank Rosenblatt's Dream and John von Neumann's Puzzle Cogn. Comput. 7, 263–278 10.1007/s12559-015-9333-0

[93] García-Jacas C.R., Pinacho-Castellanos S.A., García-González L.A. and Brizuela C.A. (2022) Do deep learning models make a difference in the identification of antimicrobial peptides? Brief. Bioinform. 23, bbac094 10.1093/bib/bbac094 35380616

[94] de Oliveira E.C.L., Santana K., Josino L., Lima e Lima A.H. and de Souza de Sales Júnior C. (2021) Predicting cell-penetrating peptides using machine learning algorithms and navigating in their chemical space. Sci. Rep. 11, 1–15 10.1038/s41598-021-87134-w 33828175 PMC8027643

[95] Manavalan B., Subramaniyam S., Shin T.H., Kim M.O. and Lee G. (2018) Machine-learning-based prediction of cell-penetrating peptides and their uptake efficiency with improved accuracy. J. Proteome Res. 17, 2715–2726 10.1021/acs.jproteome.8b00148 29893128

[96] Wei L., Xing P., Su R., Shi G., Ma Z.S. et al. (2017) CPPred-RF: a sequence-based predictor for identifying cell-penetrating peptides and their uptake efficiency. J. Proteome Res. 16, 2044–2053 10.1021/acs.jproteome.7b00019 28436664

[97] Beltran J.A., Del Rio G. and Brizuela C.A. (2020) An automatic representation of peptides for effective antimicrobial activity classification. Comput. Struct. Biotechnol. J. 18, 455–463 10.1016/j.csbj.2020.02.002 32180904 PMC7063200

[98] Fernandes F.C., Rigden D.J. and Franco O.L. (2012) Prediction of antimicrobial peptides based on the adaptive neuro-fuzzy inference system application. Biopolymers 98, 280–287 10.1002/bip.22066 23193592

[99] Wei W., Cherukupalli S., Jing L., Liu X. and Zhan P. (2020) Fsp 3: a new parameter for drug-likeness. Drug Discov. Today 25, 1839–1845 10.1016/j.drudis.2020.07.017 32712310

[100] Ohsumi Y. (2014) Historical landmarks of autophagy research. Cell Res. 24, 9–23 10.1038/cr.2013.169 24366340 PMC3879711

[101] Klionsky D.J., Abdel-Aziz A.K., Abdelfatah S., Abdellatif M., Abdoli A., Abel S. et al. (2021) Guidelines for the use and interpretation of assays for monitoring autophagy (4th edition). Autophagy 17, 1–382 10.1080/15548627.2020.1797280 33634751 PMC7996087

[102] Levine B. and Kroemer G. (2019) Biological functions of autophagy genes: a disease perspective. Cell 176, 11–42 10.1016/j.cell.2018.09.048 30633901 PMC6347410

[103] Zhang L., Dai L. and Li D. (2021) Mitophagy in neurological disorders. J. Neuroinflammation 18, 297 10.1186/s12974-021-02334-5 34937577 PMC8693476

[104] Blázquez-Bernal Á., Fernandez-Costa J.M., Bargiela A. and Artero R. (2021) Inhibition of autophagy rescues muscle atrophy in a LGMDD2 Drosophila model. FASEB J. 35, 1–19 10.1096/fj.202100539RR 34547132 PMC12316082

[105] Gorostieta-Salas E., Moreno-Blas D., Geronimo-Olvera C., Cisneros B., Court F.A. et al. (2021) Enhanced activity of exportin-1/CRM1 in neurons contributes to autophagy dysfunction and senescent features in old mouse brain. Oxid. Med. Cell. Longev. 2021, 1–22 10.1155/2021/6682336 34434486 PMC8382534

[106] Dolese D., Junot M., Ghosh B., Butsch T., Johnson A. et al. (2022) Degradative tubular lysosomes link pexophagy to starvation and early aging in C. elegans. Autophagy 18, 1522–1533 34689720 10.1080/15548627.2021.1990647 PMC9298445

[107] Liu J., Kang R. and Tang D. (2021) The art of war: ferroptosis and pancreatic cancer. Front. Pharmacol. 12, 10.3389/fphar.2021.773909 PMC8702849 34955844

[108] Fares H.M., Lyu X., Xu X., Dong R., Ding M. et al. (2021) Autophagy in cancer: the cornerstone during glutamine deprivation. Eur. J. Pharmacol. 916, 174723 10.1016/j.ejphar.2021.174723 34973953

[109] Wang M., Fan Z. and Han H. (2021) Autophagy in Staphylococcus aureus infection. Front. Cell Infect Microbiol. 11, 1–8 10.3389/fcimb.2021.750222 PMC8529010 34692566

[110] Liang W., Liu H., He J., Ai L., Meng Q. et al. (2021) Studies progression on the function of autophagy in viral infection. Front. Cell Dev. Biol. 9, 1–7 10.3389/fcell.2021.772965 PMC8716779 34977022

[111] Mizushima N. and Levine B. (2020) Autophagy in human diseases. N. Engl. J. Med. 383, 1564–1576 10.1056/NEJMra2022774 33053285

[112] Balloux F. and van Dorp L. (2017) Q&A: what are pathogens, and what have they done to and for us? BMC Biol. 15, 1–6 https://pmc/articles/PMC5648414/ 10.1186/s12915-017-0433-z 29052511 PMC5648414

[113] Martínez J.L. (2014) Short-sighted evolution of bacterial opportunistic pathogens with an environmental origin. Front Microbiol. 5, 1–4 10.3389/fmicb.2014.00239 24904552 PMC4033005

[114] Pirofski L.a. and Casadevall A. (2012) Q and A What is a pathogen? A question that begs the point BMC Biol. 10, 1–3 https://bmcbiol.biomedcentral.com/articles/10.1186/1741-7007-10-6 10.1186/1741-7007-10-6 22293325 PMC3269390

[115] Gomes L.C. and Dikic I. (2014) Autophagy in antimicrobial immunity. Mol. Cell 54, 224–233 10.1016/j.molcel.2014.03.009 24766886

[116] Colombo M.I., Gutierrez M.G. and Romano P.S. (2006) The two faces of autophagy: Coxiella and Mycobacterium. Autophagy 2, 162–164 10.4161/auto.2827 16874070

[117] Gatica D., Lahiri V. and Klionsky D.J. (2018) Cargo recognition and degradation by selective autophagy. Nat. Cell Biol. 20, 233–242 10.1038/s41556-018-0037-z 29476151 PMC6028034

[118] Saitoh T., Fujita N., Jang M.H., Uematsu S., Yang B.G. et al. (2008) Loss of the autophagy protein Atg16L1 enhances endotoxin-induced IL-1beta production. Nature 456, 264–268 10.1038/nature07383 18849965

[119] Hampe J., Franke A., Rosenstiel P., Till A., Teuber M. et al. (2007) A genome-wide association scan of nonsynonymous SNPs identifies a susceptibility variant for Crohn disease in ATG16L1. Nat. Genet. 39, 207–211 10.1038/ng1954 17200669

[120] Chovatiya R. and Medzhitov R. (2014) Stress, inflammation, and defense of homeostasis. Mol. Cell 54, 281–288 10.1016/j.molcel.2014.03.030 24766892 PMC4048989

[121] Pasupuleti M., Schmidtchen A. and Malmsten M. (2012) Antimicrobial peptides: key components of the innate immune system. Crit. Rev. Biotechnol. 32, 143–171 10.3109/07388551.2011.594423 22074402

[122] Gutierrez M.G., Master S.S., Singh S.B., Taylor G.A., Colombo M.I. et al. (2004) Autophagy is a defense mechanism inhibiting BCG and Mycobacterium tuberculosis survival in infected macrophages. Cell 119, 753–766 10.1016/j.cell.2004.11.038 15607973

[123] Subauste C.S. (2009) Autophagy as an antimicrobial strategy. Expert Rev. Anti Infect. Ther. 7, 743 https://pmc/articles/PMC3280690/ 10.1586/eri.09.41 19681702 PMC3280690

[124] Shoji-Kawata S., Sumpter R., Leveno M., Campbell G.R., Zou Z. et al. (2013) Identification of a candidate therapeutic autophagy-inducing peptide. Nature 494, 201–206 10.1038/nature11866 23364696 PMC3788641

[125] Muciño G., Castro-Obregón S., Hernandez-Pando R. and Del Rio G. (2016) Autophagy as a target for therapeutic uses of multifunctional peptides. IUBMB Life 68, 259–267 10.1002/iub.1483 26968336

[126] Xu J., Chiang H.C., Bjursell M.K. and Gordon J.I. (2004) Message from a human gut symbiont: sensitivity is a prerequisite for sharing. Trends Microbiol. 12, 21–28 10.1016/j.tim.2003.11.007 14700548

[127] Turnbaugh P.J., Ley R.E., Hamady M., Fraser-Liggett C.M., Knight R. et al. (2007) The human microbiome project. Nature 449, 804–810 10.1038/nature06244 17943116 PMC3709439

[128] Hongoh Y., Ohkuma M. and Kudo T. (2003) Molecular analysis of bacterial microbiota in the gut of the termite Reticulitermes speratus (Isoptera; Rhinotermitidae). FEMS Microbiol. Ecol. 44, 231–242 10.1016/S0168-6496(03)00026-6 19719640

[129] Greetham H.L., Giffard C., Hutson R.A., Collins M.D. and Gibson G.R. (2002) Bacteriology of the Labrador dog gut: a cultural and genotypic approach. J. Appl. Microbiol. 93, 640–646 10.1046/j.1365-2672.2002.01724.x 12234347

[130] Nagpal J. and Cryan J.F. (2021) Microbiota-brain interactions: moving toward mechanisms in model organisms. Neuron 109, 3930–3953 10.1016/j.neuron.2021.09.036 34653349

[131] Zheng D., Liwinski T. and Elinav E. (2020) Interaction between microbiota and immunity in health and disease. Cell Res. 30, 492–506 10.1038/s41422-020-0332-7 32433595 PMC7264227

[132] Bäckhed F., Ding H., Wang T., Hooper L.V., Gou Y.K. et al. (2004) The gut microbiota as an environmental factor that regulates fat storage. Proc. Natl. Acad. Sci. U.S.A. 101, 15718–15723 10.1073/pnas.0407076101 15505215 PMC524219

[133] Turnbaugh P.J. and Gordon J.I. (2009) The core gut microbiome, energy balance and obesity. J. Physiol. 587, 4153–4158 10.1113/jphysiol.2009.174136 19491241 PMC2754355

[134] DiGiulio D.B., Callahan B.J., McMurdie P.J., Costello E.K., Lyell D.J. et al. (2015) Temporal and spatial variation of the human microbiota during pregnancy. Proc. Natl. Acad. Sci. U.S.A. 112, 11060–11065 10.1073/pnas.1502875112 26283357 PMC4568272

[135] Koren O., Goodrich J.K., Cullender T.C., Spor A., Laitinen K. et al. (2012) Host remodeling of the gut microbiome and metabolic changes during pregnancy. Cell 150, 470–480 10.1016/j.cell.2012.07.008 22863002 PMC3505857

[136] Dzidic M., Boix-Amorós A., Selma-Royo M., Mira A. and Collado M.C. (2018) Gut microbiota and mucosal immunity in the neonate. Med. Sci. 6, 56 https://pmc/articles/PMC6163169/ 10.3390/medsci6030056 PMC6163169 30018263

[137] Dorschner R.A., Lin K.H., Murakami M. and Gallo R.L. (2003) Neonatal skin in mice and humans expresses increased levels of antimicrobial peptides: innate immunity during development of the adaptive response. Pediatr. Res. 53, 566–572 10.1203/01.PDR.0000057205.64451.B7 12612195

[138] Battersby A.J., Khara J., Wright V.J., Levy O. and Kampmann B. (2016) antimicrobial proteins and peptides in early life: ontogeny and translational opportunities. Front. Immunol. 7, 10.3389/fimmu.2016.00309 27588020 PMC4989132

[139] Yang X., Niu L., Pan Y., Feng X., Liu J. et al. (2020) LL-37-induced autophagy contributed to the elimination of live Porphyromonas gingivalis internalized in keratinocytes. Front. Cell Infect Microbiol. 10, 1–15 10.3389/fcimb.2020.561761 33178622 PMC7593823

[140] Hilchie A.L., Wuerth K. and Hancock R.E.W. (2013) Immune modulation by multifaceted cationic host defense (antimicrobial) peptides. Nat. Chem. Biol. 9, 761–768 10.1038/nchembio.1393 24231617

[141] Wolf M. and Moser B. (2012) Antimicrobial activities of chemokines: not just a side effect? Front. Immunol. 3, 1–12 10.3389/fimmu.2012.00213 22837760 PMC3401835

[142] Ge Y., Huang M. and Yao Y.m. (2018) Autophagy and proinflammatory cytokines: interactions and clinical implications. Cytokine Growth Factor Rev. 43, 38–46 10.1016/j.cytogfr.2018.07.001 30031632

[143] Moossavi S. and Bishehsari F. (2019) Microbes: possible link between modern lifestyle transition and the rise of metabolic syndrome. Obes. Rev. 20, 407–419 10.1111/obr.12784 30548384

[144] Trøseid M., Andersen G.Ø., Broch K. and Hov J.R. (2020) The gut microbiome in coronary artery disease and heart failure: current knowledge and future directions. EBioMedicine 52, 1–10 10.1016/j.ebiom.2020.102649 32062353 PMC7016372

[145] Elinav E., Strowig T., Kau A.L., Henao-Mejia J., Thaiss C.A. et al. (2011) NLRP6 inflammasome regulates colonic microbial ecology and risk for colitis. Cell 145, 745–757 10.1016/j.cell.2011.04.022 21565393 PMC3140910

[146] Nakamura Y., Franchi L., Kambe N., Meng G., Strober W. et al. (2012) Critical role for mast cells in interleukin-1β-driven skin inflammation associated with an activating mutation in the nlrp3 protein. Immunity 37, 85–95 10.1016/j.immuni.2012.04.013 22819042 PMC3411177

[147] Meng G., Zhang F., Fuss I., Kitani A. and Strober W. (2009) A mutation in the Nlrp3 gene causing inflammasome hyperactivation potentiates Th17 cell-dominant immune responses. Immunity 30, 860–874 10.1016/j.immuni.2009.04.012 19501001 PMC2764254

[148] Yao X., Zhang C., Xing Y., Xue G., Zhang Q. et al. (2017) Remodelling of the gut microbiota by hyperactive NLRP3 induces regulatory T cells to maintain homeostasis. Nat. Commun. 8, 1–17 10.1038/s41467-017-01917-2 29196621 PMC5711854

[149] Zong X., Fu J., Xu B., Wang Y. and Jin M. (2020) Interplay between gut microbiota and antimicrobial peptides. Anim. Nutr. (Zhongguo Xu Mu Shou Yi Xue Hui) 6, 389–396 10.1016/j.aninu.2020.09.002 PMC7750803 33364454

[150] Chen C.H. and Lu T.K. (2020) Development and challenges of antimicrobial peptides for therapeutic applications. Antibiot (Basel, Switzerland) 9, 1–20 10.3390/antibiotics9010024 PMC7168295 31941022

[151] Dijksteel G.S., Ulrich M.M.W., Middelkoop E. and Boekema B.K.H.L. (2021) Review: lessons learned from clinical trials using antimicrobial peptides (AMPs). Front. Microbiol. 12, 1–18 10.3389/fmicb.2021.616979 33692766 PMC7937881

